# Microsurgical Intervention for Dens Invaginatus in an Immature Tooth: A Case Report With Long‐Term Follow‐Up

**DOI:** 10.1155/crid/8216241

**Published:** 2025-12-17

**Authors:** Talal Al-Nahlawi, Zohreh Moradipour, Hadi Assadian

**Affiliations:** ^1^ Operative Dentistry and Endodontics Department, Syrian Private University (SPU), Damascus, Syria; ^2^ Department of Endodontics, School of Dentistry, Tehran University of Medical Sciences (TUMS), Tehran, Iran, tums.ac.ir

**Keywords:** biomaterials, cone-beam computed tomography (CBCT), dens invaginatus, dental anomaly, endodontic microsurgery, immature apex, maxillary lateral incisor, periapical abscess, root canal treatment

## Abstract

Dens invaginatus (DI) is a rare dental anomaly characterized by the infolding of enamel and dentin into the pulp cavity during tooth development. This condition often leads to complex endodontic challenges, predisposing affected teeth to caries, pulp necrosis, and chronic periapical abscesses. This case report describes the successful management of a maxillary lateral incisor with Type II DI complicated by a chronic periapical abscess, using an endodontic microsurgical approach. A 10‐year‐old girl presented with a chief complaint of recurrent swelling in the maxillary left incisor area. Clinical examination revealed a malformed maxillary left lateral incisor with Grade II mobility and a fluctuant gingival swelling. Periapical radiography and cone‐beam computed tomography (CBCT) confirmed DI with an immature apex, significant periapical rarefaction, and fenestration of the labial cortical bone. Endodontic microsurgery treatment was performed, involving flap reflection, lesion excision, root canal irrigation with 1% sodium hypochlorite (NaOCl), and retrofilling using a calcium–silicate‐based sealer (CeraSeal) and bioceramic putty (CeraPutty). The surgical site was sutured, and postoperative care instructions were provided. Histological examination confirmed a periapical cyst. Follow‐up examinations revealed favorable soft tissue healing, mucosal sinus tract closure, and complete bone regeneration at the surgical site. CBCT evaluation at 3 years confirmed full buccal bone regeneration. This case demonstrates that endodontic microsurgery can be a successful treatment option for managing complex DI cases with chronic periapical abscesses when orthograde endodontic treatment is not feasible. CBCT imaging plays a pivotal role in diagnosis, treatment planning, and postoperative assessment. Further clinical research is warranted to evaluate the long‐term outcomes of microsurgical management in similar developmental anomalies.

## 1. Introduction

Dens invaginatus (DI) is a rare developmental dental anomaly characterized by the invagination of enamel and dentin into the pulp cavity during tooth development [1, 2]. The etiology of DI remains unclear; however, several predisposing factors have been proposed, including arch constriction, enamel epithelium growth variations, enamel organ distortion, trauma, infection, or inadequate nutrition [1, 3]. DI predominantly occurs in maxillary lateral incisors, with bilateral occurrence observed in nearly half of the cases [1, 4].

First documented in a whale′s tooth by Ploquet in 1794 and later described in human dentition by Socrates in 1856, DI can range from being confined to the pulp chamber to extending into the root canal and apex [3, 5]. The prevalence of DI is estimated to range between 0.3% and 10%, predominantly involving maxillary lateral incisors [3, 5]. While clinically affected teeth may appear normal, extensive invagination often alters crown morphology, resulting in a barrel‐shaped conical structure, cingulum bifurcation, or a blind foramen on the palatal/occlusal surface [5, 6].

The abnormal canal morphology complicates endodontic management, predisposing affected teeth to caries, pulp necrosis, and chronic periapical abscesses. The fine, porous enamel and the invaginated pit provide a stagnation area for organic debris, facilitating microbial colonization [5]. Oehlers classified invaginations into three types based on apical extension of invagination and radiographic features: Type 1 represents a minor invagination confined within the crown of the tooth. Type 2 invagination penetrates into the root and forms a blind sac, without establishing communication with the periodontal ligament. Type 3 invagination is characterized by its extension through the root, establishing communication with the periodontal ligament. This type is further subdivided into two distinct subtypes: Type 3a, which occurs without pulp involvement, and Type 3b, which lacks direct pulp involvement [7].

Various treatment modalities have been proposed for DI, including both nonsurgical and surgical endodontic approaches [8–11]. Nonsurgical root canal therapy remains the preferred initial management for necrotic teeth with DI. However, the intricate and unpredictable canal morphology, presence of inaccessible spaces, and potential intracanal communications necessitate meticulous chemical and mechanical debridement, along with precise obturation techniques [3].

The selection of endodontic microsurgery for this case was based on the anatomical complexity of Type II DI and the extent of periapical involvement. While some reports have described successful management limited to the invagination in cases without pulp involvement, most cases demonstrate communication between the invagination and the pulp, increasing the risk of persistent infection and treatment failure [12]. When conventional orthograde endodontic treatment is unsuccessful or unfeasible due to complex canal morphology, microsurgical intervention offers a direct and effective approach for lesion debridement and apical sealing [12–14]. Given these considerations, endodontic microsurgery was chosen to achieve a more predictable and favorable clinical outcome.

Despite recent advances in microsurgical techniques, reports describing the management of DI‐associated chronic periapical abscesses using endodontic microsurgery remain limited. Therefore, the present case report is aimed at describing the successful management of a maxillary lateral incisor with Type II DI complicated by a chronic periapical abscess through an endodontic microsurgical approach, contributing to current literature and providing clinical insights for similar complex cases.

## 2. Case Report

### 2.1. Patient History

A 10‐year‐old girl was referred by a general dentist with a chief complaint of recurrent swelling in the maxillary left incisor area. The girl experienced pain and swelling for approximately 1 week prior to the examination, although no symptoms were present at the time of assessment. Her medical history was noncontributory. Written informed consent for both the treatment procedure and publication of the case details and clinical images was obtained from the parent prior to treatment.

### 2.2. Diagnostic Measures

**Figure 1:**
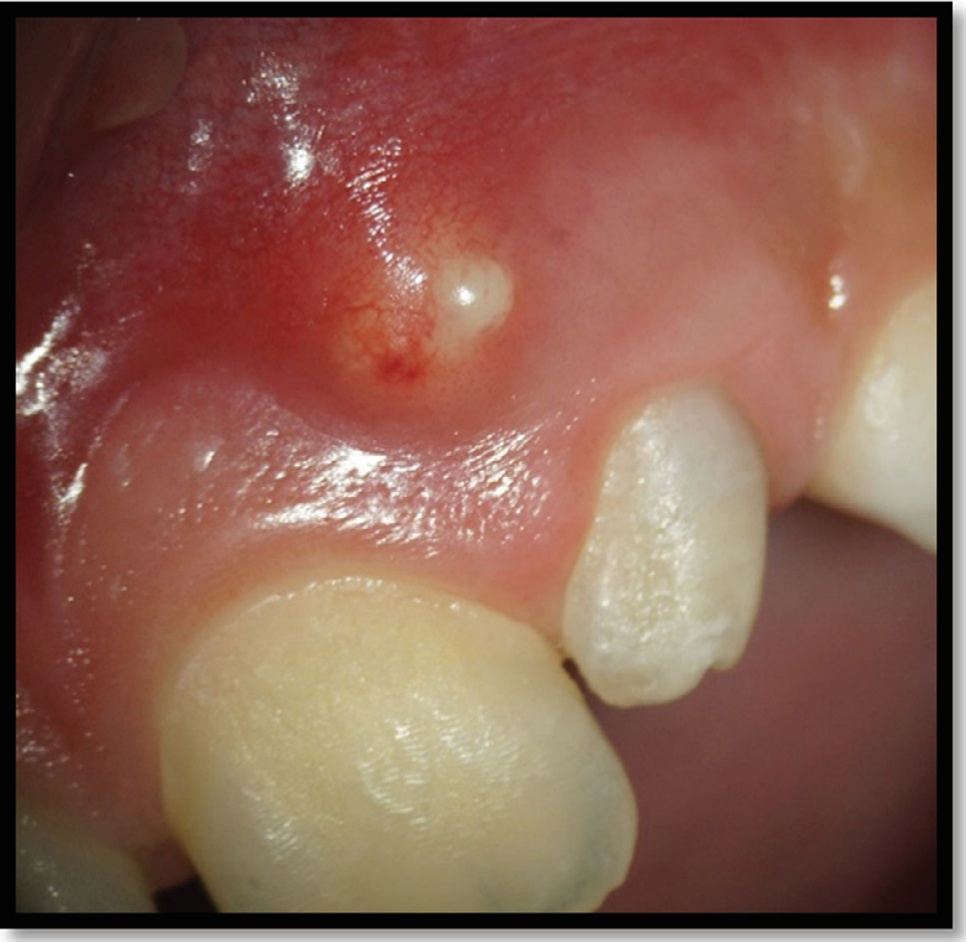
A gingival swelling due to chronic apical abscess of the maxillary left lateral incisor.

Clinical examination revealed that the maxillary left lateral incisor exhibited an anomalous crown morphology and Grade II mobility. A gingival fluctuant swelling was also identified between the maxillary left lateral and central incisors with a blocked sinus tract that subsequently drained by gentle probing pressure (Figure 1) Pulpal sensibility tests of maxillary anterior teeth indicated mere nonresponsiveness of the maxillary left lateral incisor.

Periapical radiography revealed a deep coronal enamel fissure extending from the lingual surface of the crown toward the pulp chamber of the affected tooth. Periodontal probing depths measured 2–3 mm circumferentially around the tooth, confirming the presence of a long junctional epithelial attachment at the labial aspect.

**Figure 2:**
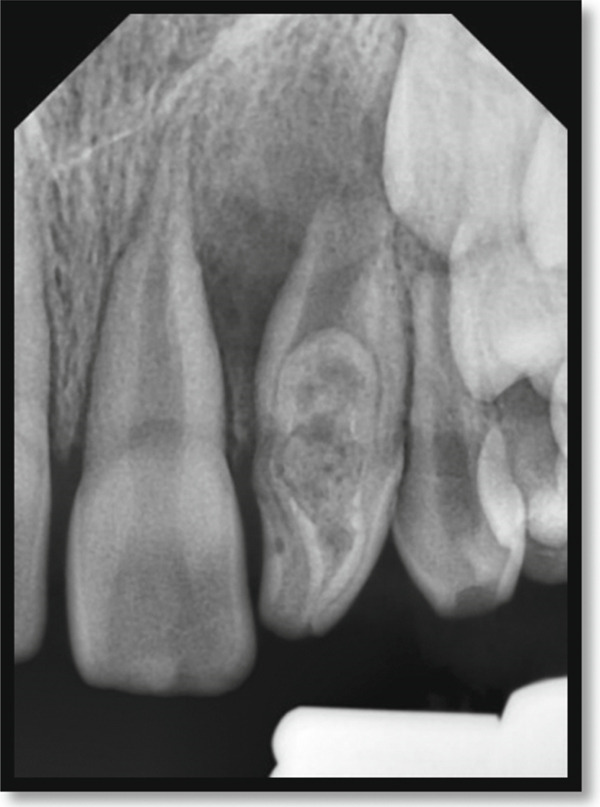
Periapical radiograph of the maxillary left lateral incisor indicating a dens invagination Type III B with a large periapical rarefaction and immature apex.

Periapical radiography confirmed DI with an immature apex and significant periapical rarefaction (Figure 2). Cone‐beam computed tomography (CBCT) was performed to obtain detailed three‐dimensional information about the extent of the lesion and surrounding structures, which could not be fully assessed on periapical radiographs. CBCT provided precise visualization of the periapical lesion and the labial cortical bone fenestration (Figure 3), aiding in accurate diagnosis and treatment planning. Although CBCT involves a higher radiation dose than conventional radiography, its use was justified in this case due to the complex anatomy, unclear boundaries of the lesion, and the need to evaluate potential involvement of adjacent teeth. The scan was performed using a limited field of view and low‐dose pediatric protocol to minimize radiation exposure, following the ALARA (as low as reasonably achievable) principle.

Figure 3CBCT image of maxillary left lateral incisor area: (a) sagittal section showing absence of the labial cortical plate and (b) axial section showing the palatal and mesial extension of the periapical lesion.

**Figure:**
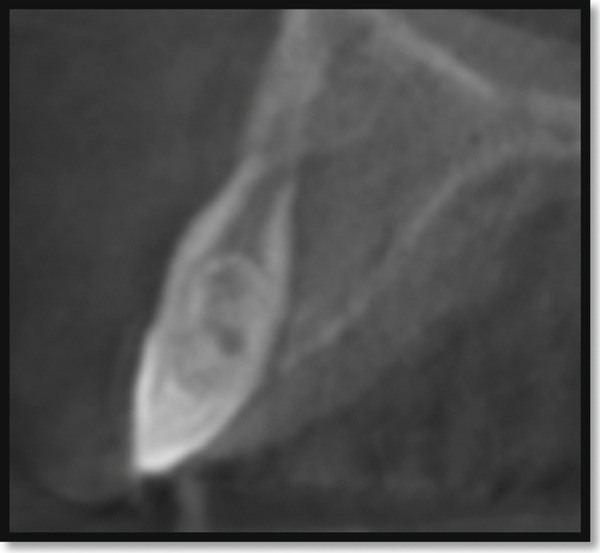
(a)

**Figure:**
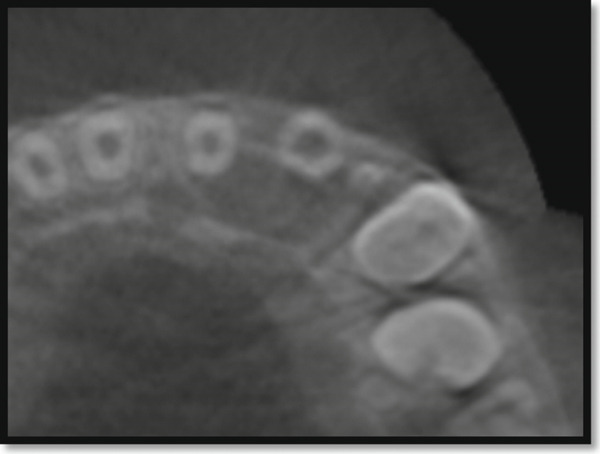
(b)

Based on the clinical, radiographic, and CBCT findings, a diagnosis of necrotic pulp with chronic periapical abscess was established for the maxillary left lateral incisor [15]. Since orthograde endodontic treatment was not feasible due to the inaccessibility of the root canal space in the orthograde approach as well as the immaturity of the root apex, endodontic microsurgery was considered as the treatment plan to save this strategic tooth in the mixed dentition stage.

### 2.3. Treatment

Endodontic microsurgery was selected over orthograde regenerative endodontic therapy due to the anatomical complexity and inaccessibility associated with Type II DI, as well as the extent of periapical bone destruction. The intricate canal morphology rendered effective orthograde disinfection—an essential factor for regenerative success—unachievable. Under these circumstances, a microsurgical approach offered a more predictable outcome by enabling direct debridement of the lesion and precise apical sealing in a structurally compromised environment.

Given the patient′s young age, behavioral management was a critical component of care. The tell–show–do (TSD) technique, combined with positive reinforcement and reassurance, was used to establish rapport and reduce treatment‐related anxiety. The procedure and instruments were explained in age‐appropriate language, allowing the patient to familiarize herself with the clinical setting, thereby enhancing cooperation during surgery.

**Figure 4:**
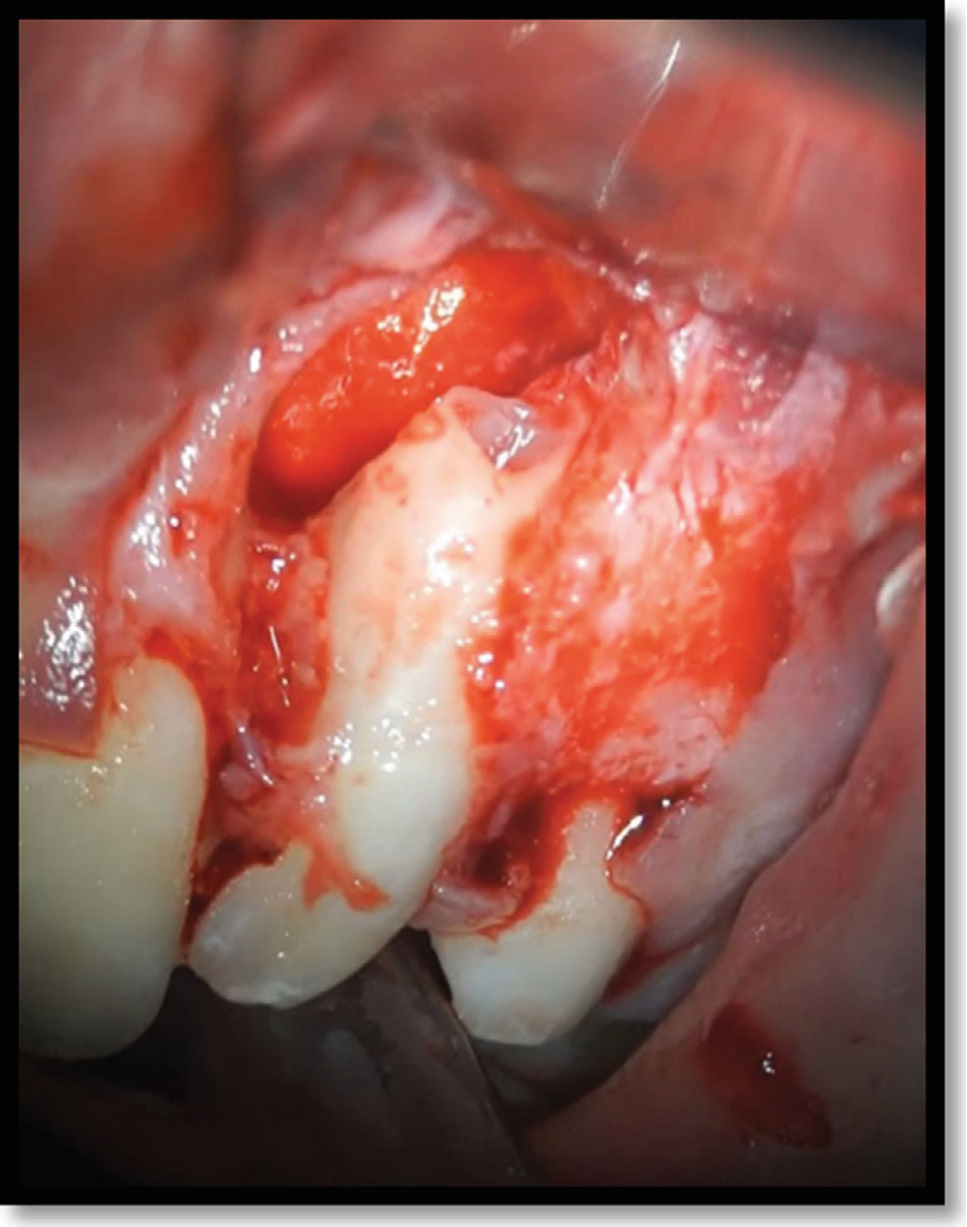
Bone dehiscence over the maxillary left lateral incisor following lesion excavation.

As the treatment was performed entirely through a surgical approach, conventional isolation methods such as rubber dam placement were not applicable. Instead, strict aseptic protocols were followed throughout the procedure. This included thorough disinfection of the surgical field, use of sterile drapes, and high‐level infection control measures to minimize contamination. Microsurgical techniques were employed under magnification to ensure precision and maintain a clean operative environment. Local anesthesia was administered via buccal and palatal infiltration. A rectangular full‐thickness mucoperiosteal flap was elevated from the mesial aspect of the maxillary left canine to the mesial aspect of the central incisor. Flap reflection revealed significant buccal cortical plate dehiscence and a large periapical lesion extending from the lateral incisor to the alveolar crest (Figure 4). The lesion was completely excised and sent for histopathological analysis.

**Figure 5:**
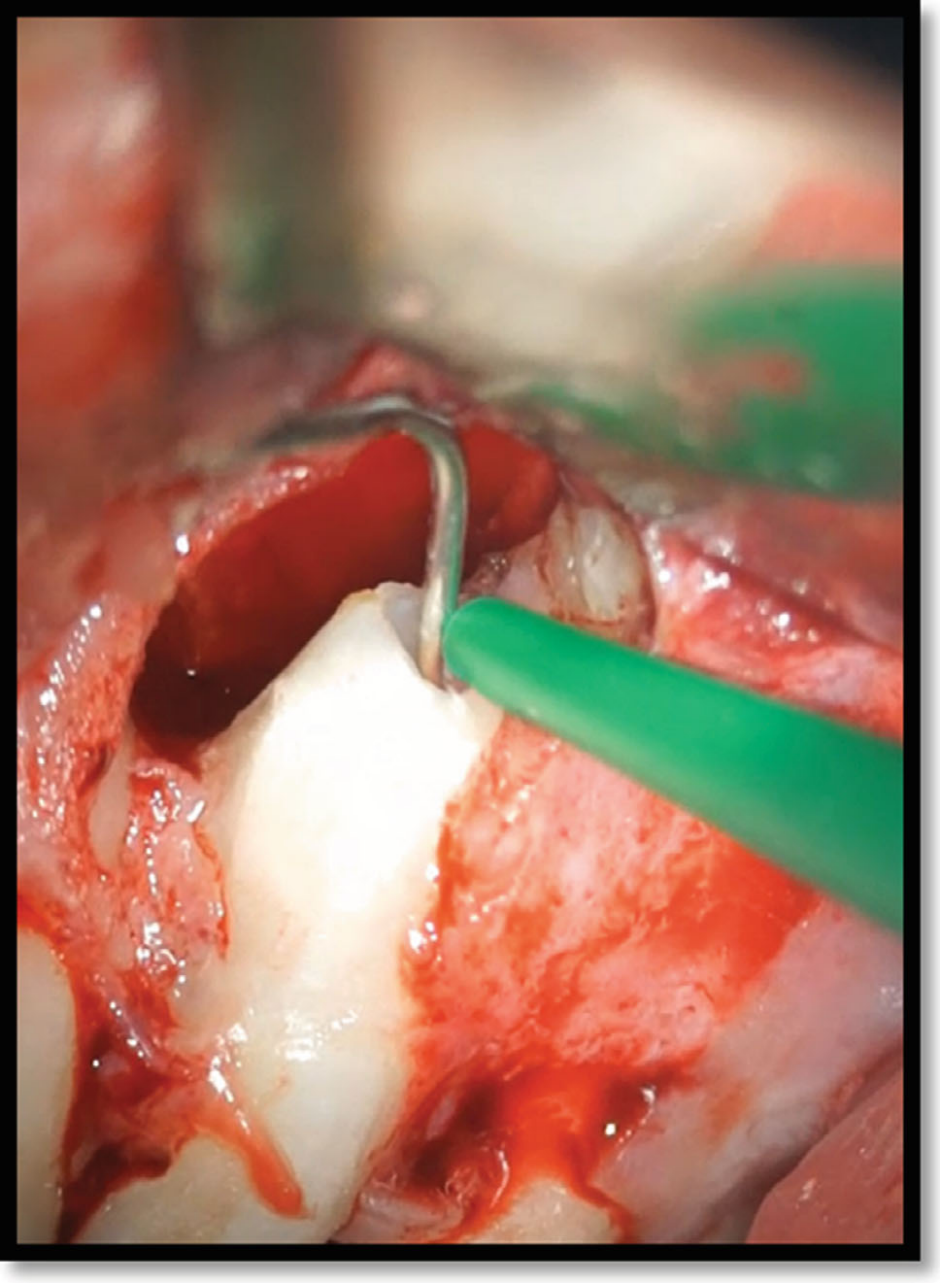
Retrograde irrigation of the root canal space using 1% NaOCl evacuated by a microsuction device.

Following lesion removal, hemostasis was achieved, and minimal apical root‐end preparation was performed (Figure 5), and the root canal was irrigated with 1% sodium hypochlorite (NaOCl), using a microsuction device for efficient irrigant removal. This facilitated the effective evacuation of the irrigant, thereby maintaining a disinfected surgical field (Figure 5).

**Figure 6:**
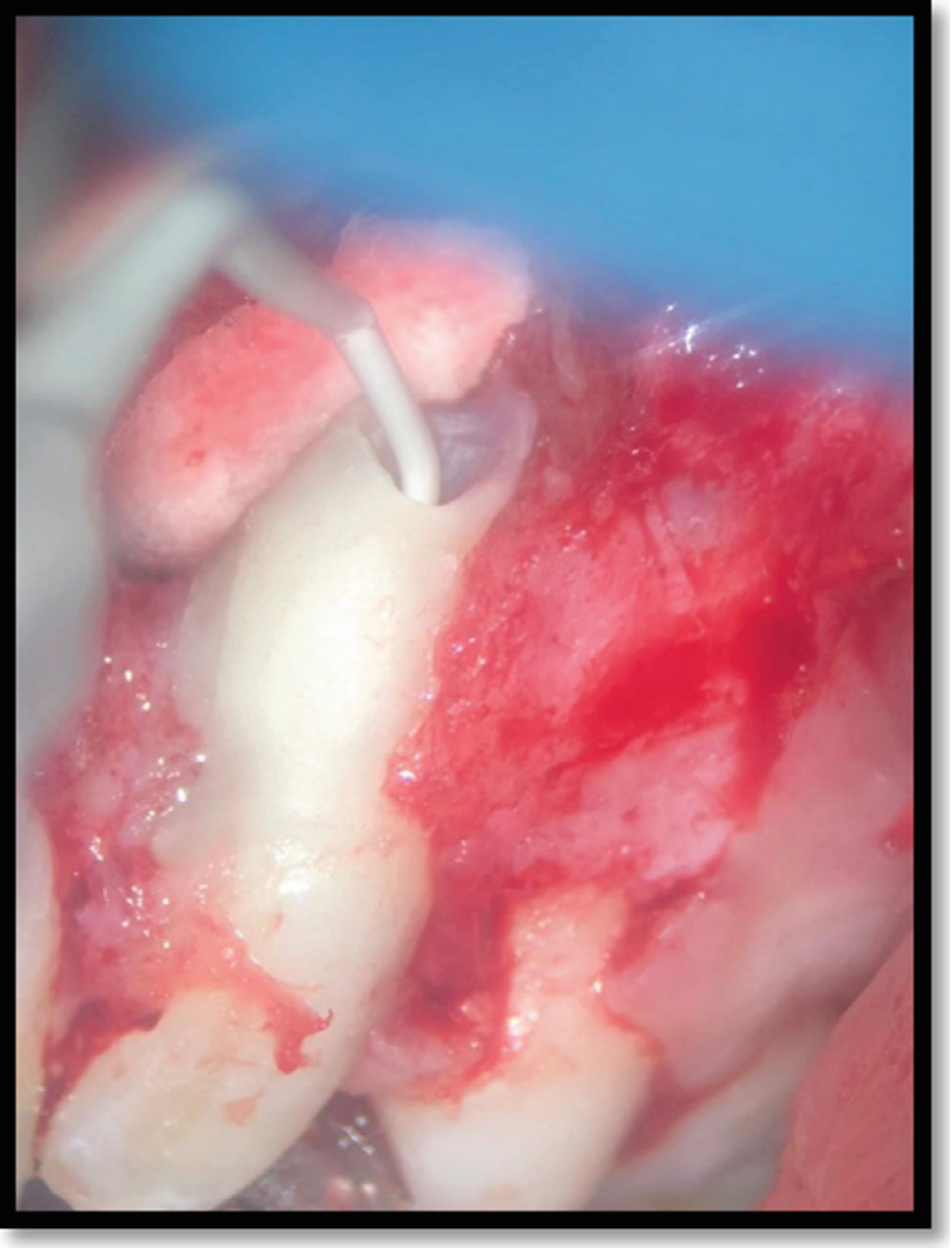
Injection of the bioceramic sealer.

**Figure 7:**
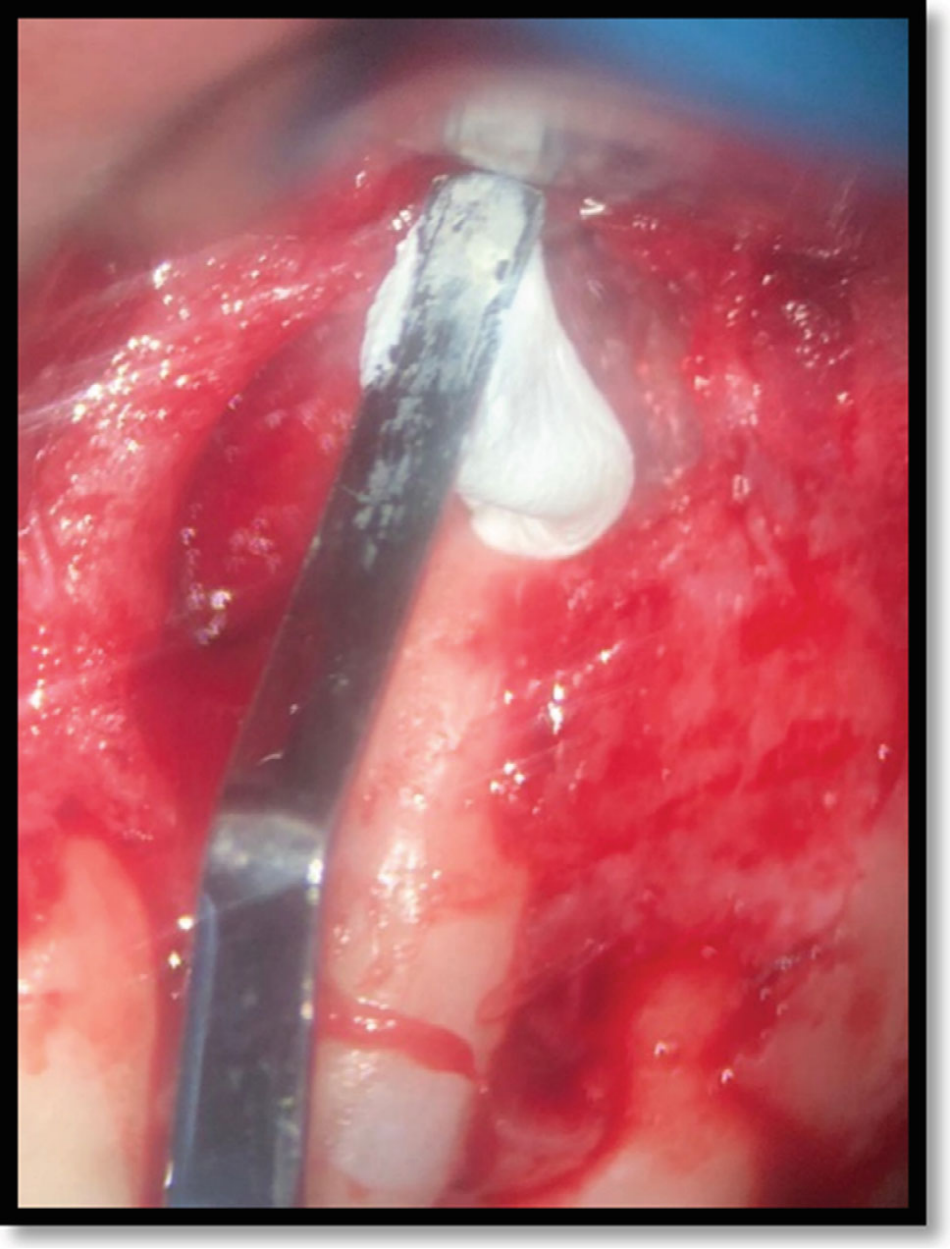
Retro‐grade application of bioceramic putty following bioceramic sealer placement.

**Figure 8:**
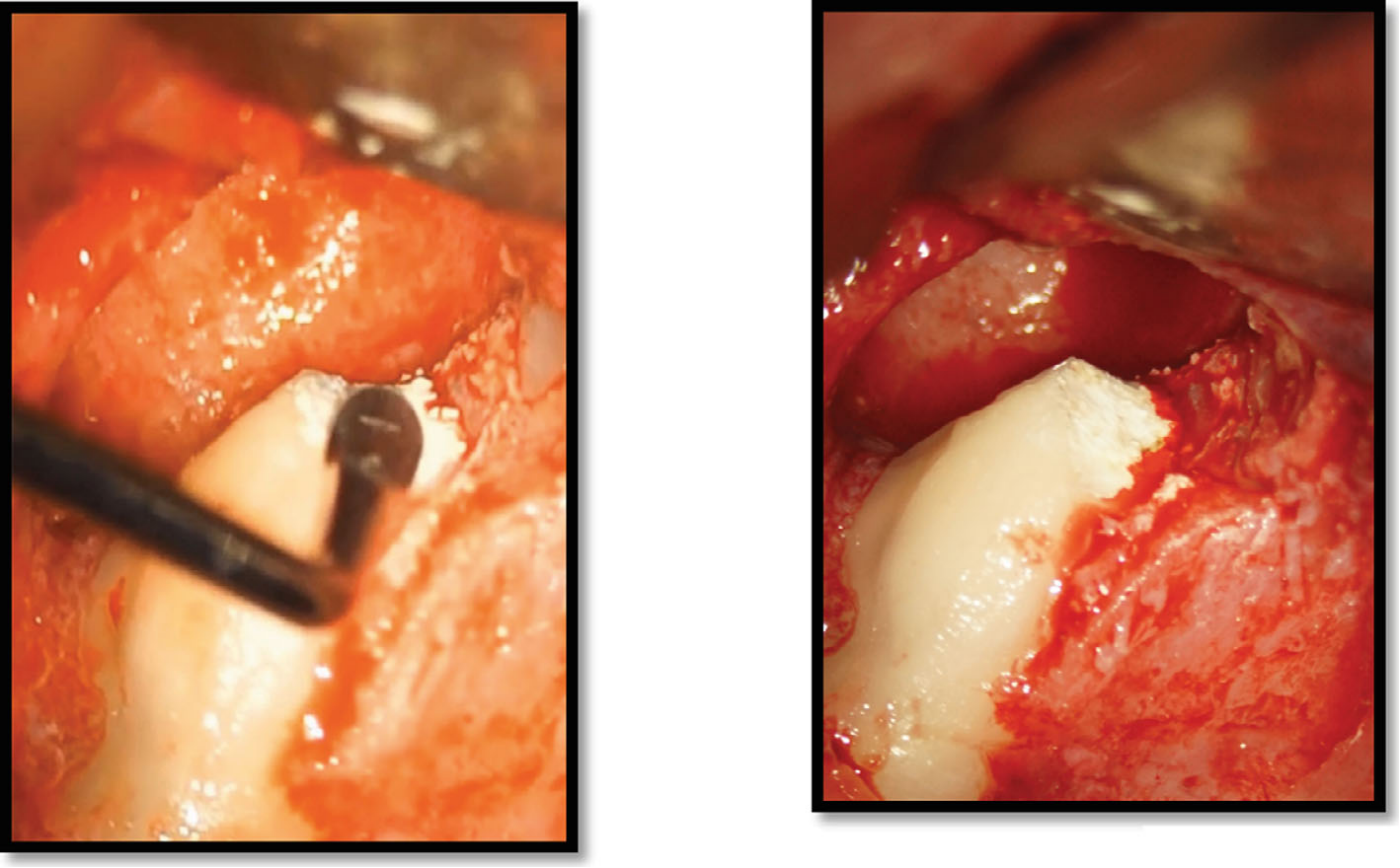
Finalized retro‐filling with bioceramic materials.

**Figure 9:**
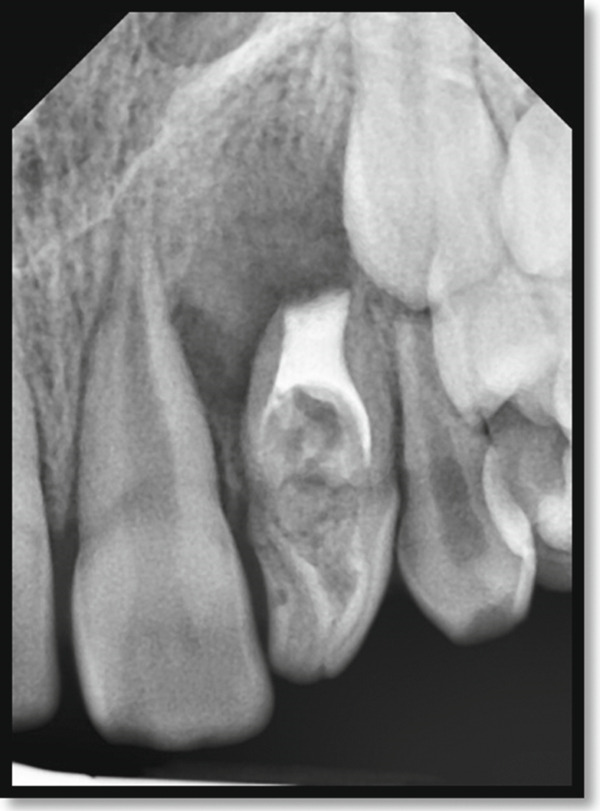
Immediate postoperative periapical radiograph indicating penetration of the bioceramic sealer into the invagination.

To prevent retrofill material displacement, sterile gauze was placed in the lesion space. The canal was dried with a Stropko Irrigator (Spartan, St. Louis, MO) before injecting a calcium–silicate‐based sealer (CeraSeal, Meta BioMed, South Korea) into the root canal (Figure 6). A manually shaped bioceramic putty (CeraPutty, Meta BioMed, South Korea) was used as a retrofilling material to seal the apex (Figure 7), with excess material removed by small excavators (Figure 8). A confirmatory periapical radiograph ensured adaptation of the retrofill material (Figure 9). Finally, the wound was closed with simple interrupted silk sutures under microscopic magnification (Zumax OMS2380, Zumax Medical, China).

**Figure 10:**
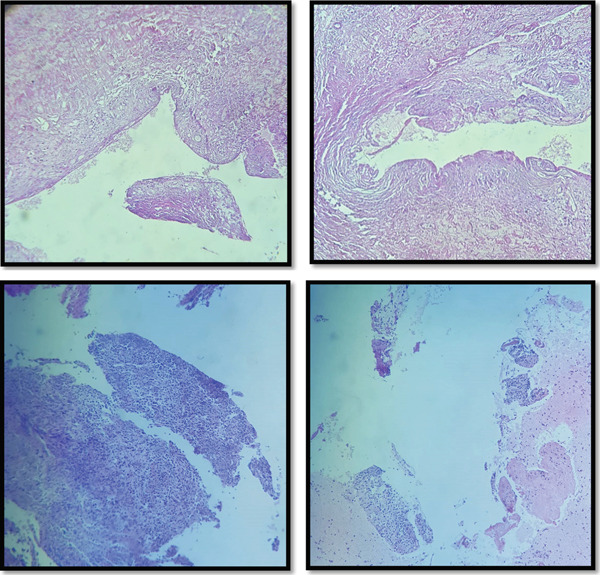
Histological examination of the excised specimen suggesting a periapical cyst.

Postoperative instructions were provided, and the patient was prescribed ibuprofen (1.2 g/d, qid for 5 days) for pain management. Histological examination confirmed a periapical cyst lined by nonkeratinizing squamous epithelium with chronic inflammatory infiltrate (Figure 10).

### 2.4. Follow‐Up

**Figure 11:**
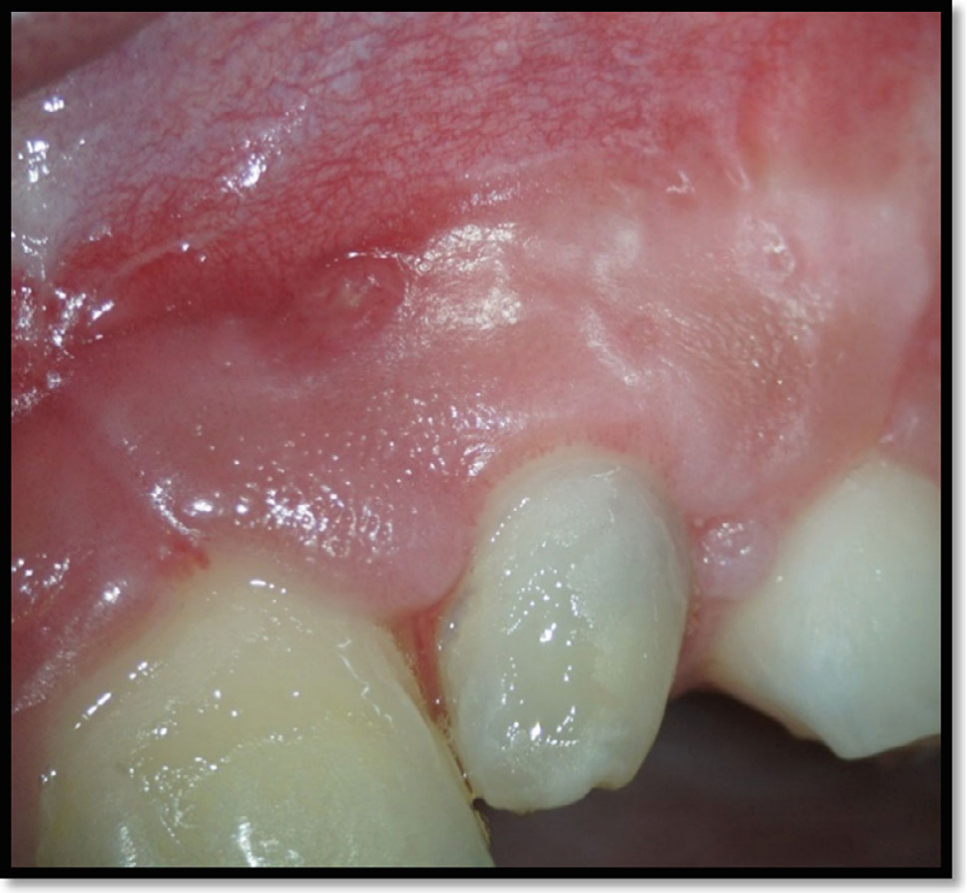
Soft tissue healing after 10 days postsurgically.

Suture removal was performed 1 week postoperatively, revealing favorable soft tissue healing without signs of inflammation or dehiscence. Closure of the mucosal sinus tract was observed by Day 10 (Figure 11). The deep enamel fissure was sealed with a preventive resin restoration using Single Bond 2 (3 M ESPE, United States) and low‐viscosity composite resin (3 M ESPE, St. Paul, MN, United States) after acid etching (Meta BioMed, South Korea).

**Figure 12:**
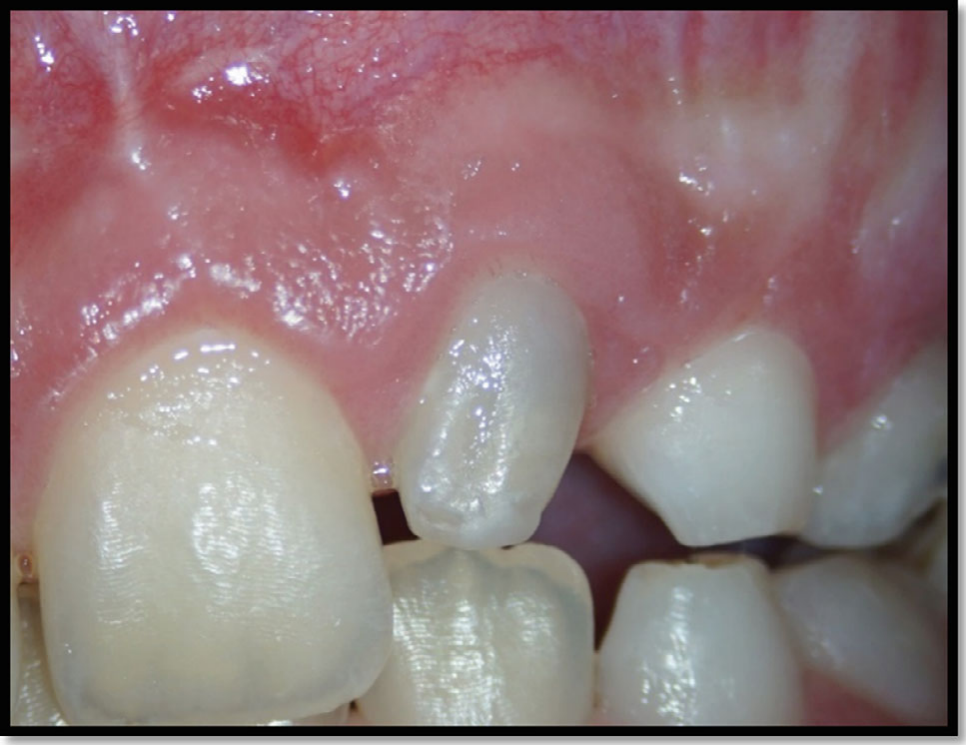
Clinical appearance of the soft tissues after 5 months of periapical surgery with very limited scar formation and minimal gingival recession.

At the 5‐month follow‐up, clinical examination showed complete soft tissue healing with minimal scarring and no significant gingival recession (Figure 12).

**Figure 13:**
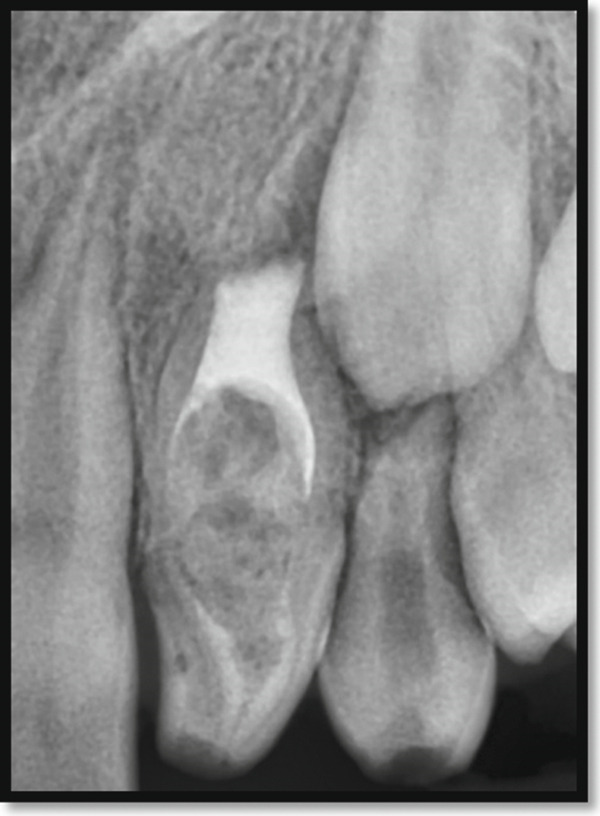
Periapical radiograph of 1‐year recall showing complete bone formation in the surgical site.

Radiographic evaluation at 1 year revealed complete bone regeneration at the surgical site, with only a minor residual radiolucency detected mesial to the unerupted canine (Figure 13). The treated tooth exhibited physiological mobility, and no pathological signs were detected.

**Figure 14:**
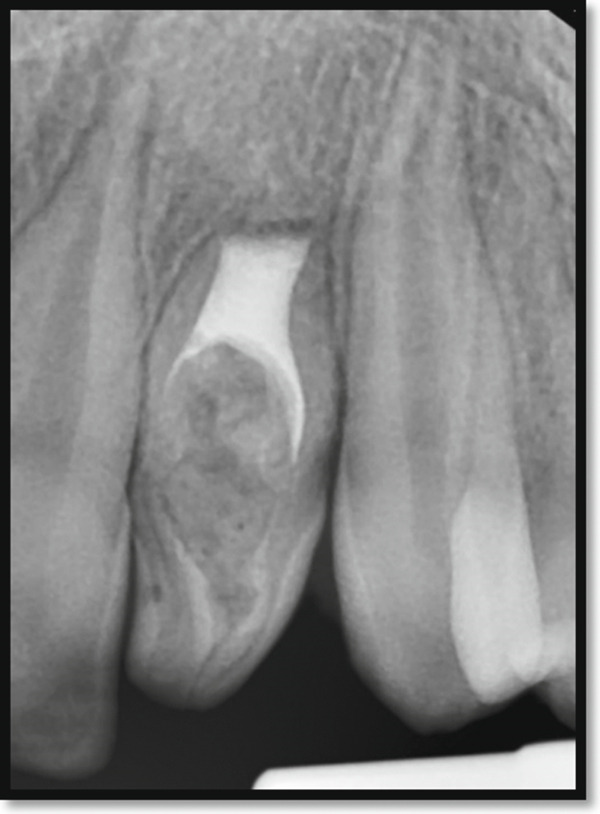
Periapical radiograph 3 years postsurgery showing complete bone healing in the area.

**Figure 15:**
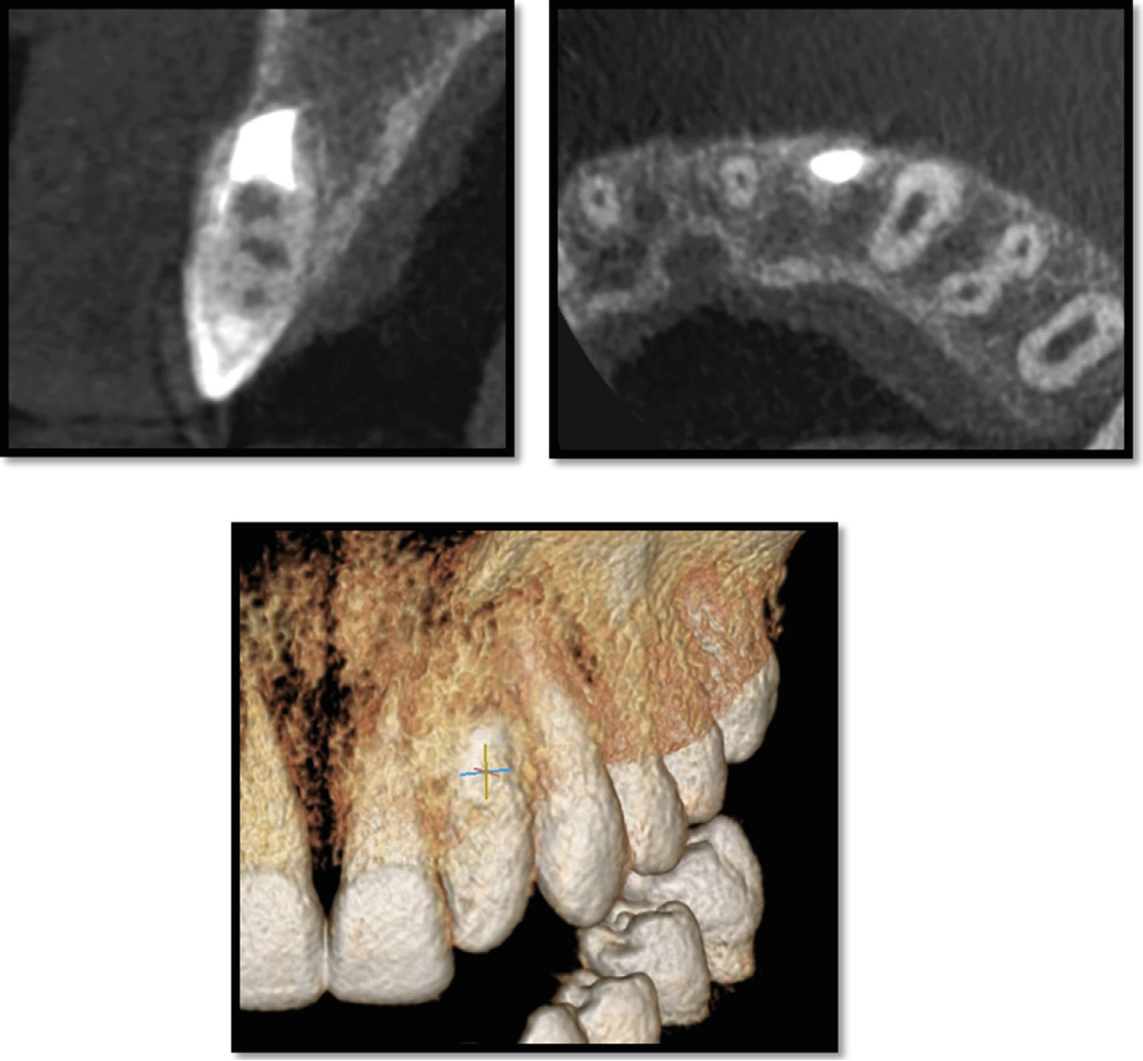
CBCT image 3 years postsurgically indicating complete bone regeneration and buccal cortical bone formation.

At the 3‐year follow‐up, clinical and radiographic findings confirmed stable function of the tooth, complete bone regeneration, and successful eruption of the ipsilateral canine (Figure 14). CBCT further demonstrated full restoration of the buccal cortical plate and complete bone regeneration at the previous lesion site (Figure 15).

The CBCT scan was performed using a field of view (FOV) of 10 × 5 cm, 85 kV, 4 mA, 8.01 s exposure time, voxel size 180 × 180 * μ*m, and a dose–area product (DAP) of 335 mGy·cm^2^. These parameters were selected to minimize radiation exposure in accordance with pediatric imaging safety standards. The examination adhered to the ALARA principle, ensuring an optimal balance between diagnostic image quality and radiation safety for the pediatric patient.

## 3. Discussion

This case report describes the successful management of a tooth diagnosed with Type II DI associated with a chronic periapical abscess using endodontic microsurgical techniques. Various treatment modalities for DI have been reported in the literature, ranging from preventive restorative approaches—such as fissure sealing of deep palatal or incisal invaginations to prevent caries development—to more invasive interventions, including nonsurgical root canal therapy, endodontic surgery, intentional replantation, and extraction [5, 16, 17]. Although nonsurgical root canal therapy remains the primary treatment option for DI with pulpal necrosis, it is often impractical in Type II and III cases due to the complex internal anatomy, which hinders effective canal cleaning and shaping without compromising structural integrity. Historically, removal of the invaginated structure was technically challenging; however, recent advancements in imaging, magnification, and microsurgical instrumentation have made such procedures more feasible and predictable [18].

In the present case, lack of structural integrity had resulted in pulpal necrosis, extension of pulpal inflammation into periapical tissues resulting in the formation of a large periapical defect destroying a large portion of the supporting alveolar and periapical bone accompanied by a chronic periapical purulent discharge and continual patient discomfort. The periapical lesion involved both maxillary lateral and central incisors; however, the ipsilateral central incisor as well as other anterior teeth responded to the pulpal sensibility tests within normal limits. Nonsurgical endodontic treatment is regarded as the primary approach for managing teeth affected by DI and pulpal necrosis.

While nonsurgical endodontic therapy is generally considered the first‐line treatment for DI, surgical management may serve as either an adjunctive or a definitive approach when orthograde access and disinfection are not achievable [11]. The introduction of modern endodontic microsurgical techniques—supported by the use of dental operating microscopes, ultrasonic instruments, and high‐magnification visualization—has markedly improved treatment outcomes. These technological advancements allow for superior illumination and precision during root‐end management, enabling thorough debridement with minimal postoperative complications.

Furthermore, the incorporation of contemporary calcium–silicate‐based materials, including premixed injectable sealers and putty‐type bioceramics with enhanced handling and sealing properties, has increased the predictability and long‐term success of microsurgical interventions [19]. In the current case, Type II DI presented a severe challenge in negotiating the root canal system. The specific morphology of the involved tooth made it impossible to remove the invaginated part through an orthograde approach and treat the remaining part endodontically. Additionally, the incomplete formation of the root apex further complicated the treatment.

Despite the patient′s young age, endodontic microsurgery provided a conservative and effective means of achieving infection control and periapical bone regeneration. This approach aligns with findings by Yang et al., who reported consistently high success rates and favorable long‐term outcomes in complex DI cases managed with microsurgical techniques [20].

Conversely, Zhang et al. have identified certain limitations of endodontic microsurgery as a standalone treatment [21]. Endodontic surgery is generally contraindicated in teeth presenting with an unfavorable crown‐to‐root ratio, compromised periodontal support, inadequate root canal obturation, or deficient coronal restorations. Additional factors influencing treatment planning include surgical accessibility, local anatomical considerations, and the patient′s overall ability to tolerate lengthy and complex procedures [22].

Apical root resection is typically performed during endodontic microsurgery to remove apical ramifications [23, 24]. However, in the present case, apical resection was intentionally omitted due to incomplete root formation, which could have further compromised the crown‐to‐root ratio. Additionally, the interproximal extension of the periradicular lesion between the maxillary central and lateral incisors facilitated the excision of the lesion without necessitating root‐end resection. Furthermore, the blunderbuss morphology of the apical root allowed for root‐end preparation and retro‐filling using calcium‐silicate materials.

The use of calcium–silicate‐based biomaterials is now regarded as the standard of care for root‐end fillings in endodontic microsurgery due to their superior biocompatibility, dimensional stability, and sealing ability [25]. To optimize the adaptation of the retrograde filling material, the “Lid technique,” as described by Enkhbileg et al. [26], was employed. Initially, an injectable premixed calcium–silicate‐based sealer was inserted into the prepared retro‐cavity. Subsequently, a putty‐like bioceramic was added using a microsurgical curette to exert hydraulic forces, ensuring the material was pushed as deeply as possible into the irregularities.

Previous investigations have underscored the efficacy of calcium hydroxide as an intracanal medicament in the disinfection of apical periodontitis associated with Type II DI [27–29]. A recent case series reported successful outcomes in four patients managed using calcium hydroxide, although none of the cases exhibited the complex canal morphology involving five root canals, as observed in the present case [29]. The presence of large periapical lesions, as seen in both past studies and this case, further reinforces the antimicrobial efficacy of calcium hydroxide against high microbial loads [30, 31].

A recent comparative study evaluating nanoleakage in retrograde fillings demonstrated that calcium–silicate‐based putty materials, particularly when applied using the Lid technique, achieved superior sealing ability and required less procedural time compared to mineral trioxide aggregate (MTA) [26].

Although the use of bone grafting materials and collagen membranes for guided tissue regeneration has been well documented to enhance bone repair and limit epithelial proliferation in large apicomarginal defects [32], this adjunctive approach was not implemented in the present case due to financial constraints. Such regenerative procedures are particularly beneficial in cases where the periosteum is compromised by sinus tract formation or chronic infection [33]. By preventing epithelial cell proliferation, the use of guided tissue regeneration can lead to more effective and lasting bone regeneration [34]. However, despite these advantages, the economic constraints faced by the patient necessitated the omission of this otherwise beneficial treatment option.

Several studies have reported the management of Type II DI in maxillary lateral incisors, highlighting the challenges posed by complex canal anatomy and periapical pathology. For instance, Tsurumachi et al. described successful nonsurgical endodontic treatment in a case with limited periapical involvement [35], whereas cases with extensive lesions or inaccessible invaginations often required surgical intervention [16]. Microsurgical approaches, as reported by Sübay and Kayataş provided predictable outcomes in cases where conventional orthograde treatment was not feasible, allowing direct lesion debridement and precise apical sealing [36]. In comparison, the present case demonstrates the successful long‐term management of a Type II DI associated with chronic periapical abscess through endodontic microsurgery, confirmed radiographically and via CBCT at 1‐ and 3‐year follow‐ups. The integration of contemporary bioceramic materials (CeraSeal and CeraPutty) further supports favorable healing and contributes to the growing body of evidence on advanced management strategies for complex DI cases.

The choice of CeraSeal and CeraPutty over the more commonly used MTA was based on their favorable handling properties, enhanced biocompatibility, and excellent sealing ability. Unlike MTA, which has limitations such as difficult manipulation, potential discoloration, and prolonged setting time, these newer bioceramic materials offer improved injectability, dimensional stability, and faster clinical application. In addition, both CeraSeal and CeraPutty release calcium ions that promote mineralization and periapical healing, while exhibiting favorable microleakage and adaptation to dentinal walls compared to MTA, as supported by recent studies [37, 38]. These properties make them particularly suitable for achieving a reliable apical seal in anatomically complex cases.

The successful outcome in this case underscores the importance of advanced imaging techniques such as the use of CBCT in accurate and detailed diagnosis of DI, particularly in cases complicated by chronic periapical pathology. The use of CBCT in endodontic diagnosis of complicated cases is well supported by the American Association of Endodontists and the European Endodontic Society in cases requiring advanced diagnostic clarity, such as complex root canal anatomy, contradictory signs and symptoms, assessment of endodontic treatment complications, and presurgical evaluation for complex periradicular surgery [39]. In this case, CBCT provided essential three‐dimensional visualization, facilitating comprehensive evaluation of the invagination, assessment of pulpal‐periodontal communications, and precise delineation of periapical bone destruction. These diagnostic insights enabled an effective and targeted treatment strategy [40]. Previous studies similarly highlight CBCT as an indispensable diagnostic adjunct in both surgical and nonsurgical management of complicated DI, enhancing preoperative planning and clinical outcomes [41].

DI, although not directly involving the pulpal anatomy, significantly disrupts the overall dental structure. The invagination of enamel into dentin creates a cavity that facilitates the accumulation and proliferation of organic material and oral bacteria. The delicate and porous enamel lining of DI allows for microbial infiltration through the infected pits, potentially progressing to dental caries or extensive involvement of the pulpal space [7, 42, 43]. The chronic periapical abscess observed in this case was likely a consequence of such abnormal morphology associated with DI, predisposing the tooth to pulp necrosis [43, 44]. The complex canal anatomy likely contributed to persistent infection and limited the feasibility of conventional or preventive approaches. Nevertheless, existing evidence suggests that the elimination of etiological factors and the establishment of an effective apico‐coronal seal can promote healing and regeneration of periradicular tissues, even in teeth with intricate canal morphology [45, 46]. In this case, meticulous debridement and apical sealing achieved through microsurgery resulted in complete healing during follow‐up.

Performing endodontic microsurgery in pediatric or adolescent patients presents additional challenges, primarily due to variable compliance and limited tolerance for lengthy procedures. The clinician′s microsurgical proficiency, together with access to appropriate magnification systems and instruments, plays a pivotal role in achieving successful outcomes. Despite the favorable results observed in this case, the absence of long‐term follow‐up represents a limitation, restricting conclusions regarding treatment durability. Furthermore, as a single‐case report, the evidence level remains low, limiting the generalizability of the findings. Future research should therefore focus on prospective studies with larger sample sizes and extended follow‐up periods to validate the clinical efficacy of endodontic microsurgery in DI cases. The development of standardized treatment protocols is also recommended to enhance clinical consistency, facilitate training, and support evidence‐based decision‐making in the management of complex endodontic anomalies.

While microsurgical endodontics offers substantial benefits in terms of precision and success rate, it also presents certain limitations. Successful outcomes depend heavily on clinician expertise, specialized training, and access to advanced equipment. The high cost of microsurgical instruments, magnification systems, and bioceramic materials may further limit accessibility in resource‐constrained settings. Moreover, long‐term durability remains contingent upon factors such as apical sealing integrity, microbial control, and biomechanical stability. Continued research is needed to optimize these parameters, refine techniques, and expand the accessibility of microsurgical care.

## 4. Conclusion

In conclusion, this case highlights that even highly complex presentations of Type II DI, complicated by chronic periapical abscess and immature root formation, can be successfully managed through meticulous diagnosis, precise case selection, and the use of contemporary endodontic microsurgical techniques. The favorable clinical and radiographic outcomes achieved over a 3‐year follow‐up demonstrate that microsurgical intervention—when combined with advanced imaging modalities and bioactive sealing materials—can effectively preserve teeth that would otherwise be deemed untreatable.

Beyond its clinical success, this report reinforces the transformative role of modern bioceramic materials and CBCT‐guided microsurgery in addressing anatomic complexities that challenge conventional endodontic therapy. The case underscores a paradigm shift in the management of DI—from extraction or non‐surgical limitations toward biologically conservative, regenerative, and tooth‐preserving microsurgical strategies.

Future research involving larger sample sizes and long‐term follow‐up is essential to establish standardized protocols and strengthen the evidence base for microsurgical treatment of developmental endodontic anomalies. Through continued integration of imaging, biomaterials, and minimally invasive surgical innovations, endodontic microsurgery holds the potential to redefine the limits of tooth preservation and functional rehabilitation in complex cases.

## Conflicts of Interest

None of the authors have a conflict of interest to disclose.

## Funding

No funding was received for this manuscript.

## Data Availability

The data that support the findings of this study are available from the corresponding author upon reasonable request.
